# Luteolin and Apigenin Attenuate 4-Hydroxy-2-Nonenal-Mediated Cell Death through Modulation of UPR, Nrf2-ARE and MAPK Pathways in PC12 Cells

**DOI:** 10.1371/journal.pone.0130599

**Published:** 2015-06-18

**Authors:** Pei-Shan Wu, Jui-Hung Yen, Mei-Chun Kou, Ming-Jiuan Wu

**Affiliations:** 1 Department of Biotechnology, Chia Nan University of Pharmacy and Science, Tainan, 717, Taiwan; 2 Department of Molecular Biology and Human Genetics, Tzu Chi University, Hualien, 970, Taiwan; Taipei Medical University, TAIWAN

## Abstract

Luteolin and apigenin are dietary flavones and exhibit a broad spectrum of biological activities including antioxidant, anti-inflammatory, anti-cancer and neuroprotective effects. The lipid peroxidation product 4-hydroxy-2-nonenal (4-HNE) has been implicated as a causative agent in the development of neurodegenerative disorders. This study investigates the cytoprotective effects of luteolin and apigenin against 4-HNE-mediated cytotoxicity in neuronal-like catecholaminergic PC12 cells. Both flavones restored cell viability and repressed caspase-3 and PARP-1 activation in 4-HNE-treated cells. Luteolin also mitigated 4-HNE-mediated LC3 conversion and reactive oxygen species (ROS) production. Luteolin and apigenin up-regulated 4-HNE-mediated unfolded protein response (UPR), leading to an increase in endoplasmic reticulum chaperone GRP78 and decrease in the expression of UPR-targeted pro-apoptotic genes. They also induced the expression of Nrf2-targeted HO-1 and xCT in the absence of 4-HNE, but counteracted their expression in the presence of 4-HNE. Moreover, we found that JNK and p38 MAPK inhibitors significantly antagonized the increase in cell viability induced by luteolin and apigenin. Consistently, enhanced phosphorylation of JNK and p38 MAPK was observed in luteolin- and apigenin-treated cells. In conclusion, this result shows that luteolin and apigenin activate MAPK and Nrf2 signaling, which elicit adaptive cellular stress response pathways, restore 4-HNE-induced ER homeostasis and inhibit cytotoxicity. Luteolin exerts a stronger cytoprotective effect than apigenin possibly due to its higher MAPK, Nrf2 and UPR activation, and ROS scavenging activity.

## Introduction

**Fig 1:**
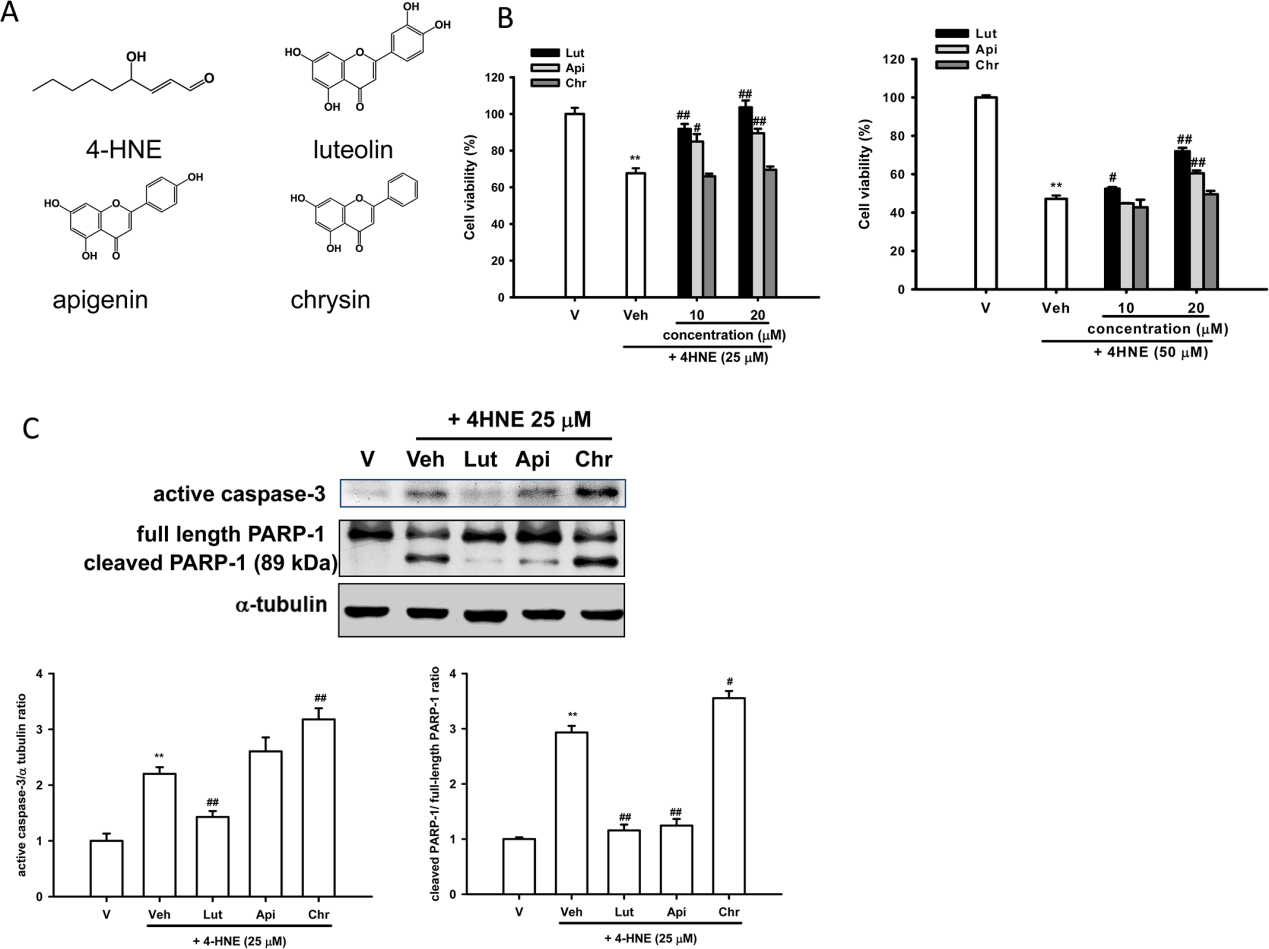
Effects of luteolin, apigenin and chrysin on 4-HNE-mediated cell death, caspase-3 activation and PARP-1 proteolysis in PC12 cells. (**A**) Structures of 4-HNE, luteolin, apigenin and chrysin (**B**) Effects of luteolin, apigenin and chrysin on 4-HNE-mediated cytotoxicity. PC12 cells were treated with luteolin, apigenin and chrysin (10 and 20 μM) 30 min prior to 4-HNE (25 and 50 μM) treatment for 16 h at 37x. Cell viability was measured by MTT as described in Materials and Methods. (**C**) Western blot analysis of active caspase-3 and PARP-1 activation. Cell lysates prepared from those treated with 4-HNE and indicated reagent for 6 h were subjected to SDS-PAGE and then were immunoblotted with antibodies that recognize cleaved caspase-3, PARP-1 and α-tubulin. The blots are representative from one of three independent experiments. Data obtained from immunoblots were then analyzed using Phoretix Gel Analysis Software as described under Materials and methods. Data represent the mean ± SD of three independent experiments. **, *p*<0.01 represents significant differences compared with vehicle control (without 4-HNE). #, *p*<0.05; ##, *p*<0.01 represent significant differences compared with 4-HNE-treated vehicle group.

4-Hydroxy-2-nonenal (4-HNE) (**Fig 1A**) is the major type of 4-hydroxyalkenals end product, generated by decomposition of arachidonic acid and larger PUFAs, through enzymatic or non-enzymatic processes [1]. It is a highly reactive electrophilic molecule and readily attacks nucleophilic centers in proteins, phospholipids, and nucleotides [1]. Under oxidative stress, its cellular concentration accumulates up to concentrations of 10 μM-5 mM from 0.1–0.3 μM of the normal range [1,2]. There is compelling evidence that 4-HNE is highly elevated in neurofibrillary tangles and senile plaques in Alzheimer’s disease (AD), in the cytoplasm of the residual motor neurons in sporadic amyotrophic lateral sclerosis (ALS), and in Lewy body in Parkinson’s disease (PD) or diffuse Lewy body disease (DLBD) [3,4].

Endoplasmic reticulum (ER) stress is one of the major adaptive signaling pathways activated by 4-HNE [5]. To cope with ER stress, cells activate unfolded protein response (UPR), a series of events that serve to re-establish ER homeostasis [6,7]. Recent research reveals that 4-HNE treatment causes upregulation of UPR in various kinds of cells [5,8–11]. Our group also reported that 4-HNE treatment triggers three canonical pathways of UPR, namely IRE1 (inositol-requiringprotein1)-XBP1 (X-box-binding protein 1), ATF6 (activating transcription factor 6) and PERK (protein kinase RNA-like ER kinase)-eIF2α (eukaryotic translation initiation factor 2α)-ATF4 (activating transcription factor 4), and induces the expression of UPR-targeted genes in neuronal-like catecholaminergic PC12 cells [12].

4-HNE is a very effective activator of Keap1-Nrf2 (NF-E2-related factor 2) and induces the expression of ARE (antioxidant-response element)-mediated enzymes [13]. In an earlier work, we reported that 4-HNE strongly upregulates Nrf2-mediated heme oxygenase (HO)-1 and glutamate-cysteine ligase catalytic subunit (GCLC) expression downstream of ER stress in PC12 cells [12]. Nrf2 also controls the expression of the xCT subunit of system Xc^₋^, which is an amino acid antiporter mediating the exchange of extracellular L-cystine and intracellular L-glutamate across the cellular plasma membrane [14].

ER stress and UPR signaling also activate stress-activated protein kinases (SAPKs), including NH_2_-terminal kinase (JNK) [15], and p38 MAPK [16]. Both signaling pathways are considered to be detrimental, and participate in the development of ER stress-related pathologies. However, SAPK signaling has positive effects on ER homeostasis via the regulation of UPR signaling elements [17]. There are also reports on the pro-survival roles for mammalian p38 MAPK in response to oxidative stress, which involves an up-regulation of antioxidant defenses [18,19]. Following this line, we found that 4-HNE-induced p38 MAPK activation may serve as an upstream negative regulator of ER stress, and confer adaptive cytoprotection against 4-HNE-mediated cell injury [12].

Flavonoids are a variety of polyphenolic compounds widely distributed in plants that are regularly consumed in an ordinary diet. Flavonoids are generally safe and can cross the blood-brain barrier (BBB), making them ideal candidates for the use in the prevention of neurodegenerative disorders [20]. Although flavonoids are still mistakenly regarded as simply acting as antioxidants, recent evidence suggests that their beneficial effects involve the modulation of stress signaling, increases in protective signaling and neurohormetic effects [21]. Luteolin and apigenin are flavones that differ in their number of hydroxyl groups in the B ring (**Fig 1A**). They have multiple bioactive and neuroprotective effects, and exhibit anti-inflammatory activities in microglia [22–24]. Luteolin attenuates the neurotoxicities induced by peroxide [25], amyloid β (Aβ) protein [26] and 6-OHDA [27–29] in cell culture. *In vivo* treatment with luteolin protected mice brain from traumatic brain injury by inhibiting inflammatory response and inducing autophagy [30]. Apigenin also protected rat and mice neurons against Aβ-induced neurotoxicity [31,32]. Although the biological functions of luteolin and apigenin are well-known, few studies have investigated their effects to protect neuronal-like catecholaminergic PC12 cells from 4-HNE-mediated cytotoxicty with a special focus on UPR and hormetic cellular stress-response pathways involved.

## Materials and Methods

### Chemicals

4-Hydroxy-trans-2-nonenal (4-HNE) was purchased from Cayman Chemical (Ann Arbor, MI). Luteolin (≥98%), apigenin (≥97%), chrysin (≥97%), MTT (3-[4, 5-dimethylthiazol-2-yl]-2, 5-diphenyl tetrazolium bromide), RPMI-1640 medium, H_2_O_2_, N-acetyl-cysteine (NAC), tunicamycin (Tm), protein kinase A (PKA) inhibitor H-89 [N-[2-((p-Bromocinnamyl)amino)ethyl]-5-isoquinolinesulfonamide] and other chemicals were purchased from Sigma-Aldrich Co. (St. Louis, MO), unless otherwise indicated. SB 203580 (4-[4'-fluorophenyl]- 2-[4'-methylsulfinylphenyl]-5-[4'-pyridyl] imidazole), a p38 MAP kinase inhibitor was purchased from Promega (Madison, WI). MEK inhibitor U0126, and c-Jun NH_2_-terminal kinase (JNK) inhibitor SP600125 (anthra[1,9-*cd*]pyrazole-6 (2*H*)-one) were purchased from Calbiochem (San Diego, CA).

### PC12 Cell Culture and Drug Treatment

The rat adrenal pheochromocytoma cell line PC12 was obtained from the Bioresource Collection and Research Center (Hsinchu, Taiwan) and maintained in RPMI-1640 medium, which contains 2 mM glutamine, 1.5 g/l sodium bicarbonate, 4.5 g/l glucose, 10 mM HEPES, 1 mM sodium pyruvate, 100 U/ml penicillin and streptomycin, supplemented with 10% heat-inactivated horse serum (Hyclone, Logan, UT) and 5% fetal bovine serum (Invitrogen, Carlsbad, CA) in 5% CO_2_ incubator at 37°C. For experiments testing the ability of 4-HNE to induce cytotoxicity, cells were incubated in serum-free RPMI medium in the absence or presence of 4-HNE [33,34].

### Drug Treatments and Cell Viability Assay

PC12 cells (1×10^6^/ml) were seeded in 6-well plates in serum-free RPMI-1640 medium and pretreated with the indicated reagent or an equivalent volume of DMSO vehicle (final concentration of 0.1%) for 30 min, followed by 4-HNE (25 μM) for 16 h. Cell viability was assessed by the mitochondrial-dependent reduction of 3-(4, 5-dimethylthiazol-2-yl)-2, 5-diphenyl tetrazolium bromide (MTT) to purple formazan [35] and Calcein AM (Invitrogen) [36]. Briefly, cells were incubated with 5 μM Calcein AM for 30 min at 37°C, and the fluorescent signal was monitored using 485 nm excitation and 530 nm emission wavelengths.

### Protein Extraction and Immunoblotting

Total cell lysates and nuclear extracts were prepared using ice cold RIPA buffer (Thermo Fisher Scientific, Inc., Rockford, IL) and Nuclear Extraction Kit (Cayman), respectively. The protein concentration was measured by the Bradford method (Bio-Rad Laboratories, Hercules, CA, USA) using bovine serum albumin as a standard. Cell lysates were separated on 8–12% SDS-PAGE and transferred onto Hybond ECL nitrocellulose (GE Healthcare) at 20 volt overnight at 4°C. The membranes were blocked at 4°C in PBST blocking buffer (5% BSA in PBS with 0.05% Tween 20, pH 7.4) for 8 h. Blots were analyzed with each antibody (**Table 1**) at a dilution of 1:1000 overnight at 4°C. After three washes with PBST, the blots were incubated with suitable horseradish peroxidase-conjugated secondary antibody (Jackson ImmunoResearch, West Grove, PA) at a dilution of 1:10,000–25,000 for 1 h. The blots were washed again and the proteins of interest were detected by Amersham ECL Prime Western Blotting Detection Reagents (GE Healthcare) according to the manufacturer’s instructions, and the chemiluminescence signal was then visualized with X-ray film. For reprobing, blots were treated with Restore stripping solution (Thermo Scientific). The intensities of these bands were analyzed with Phoretix Gel Analysis Software (Nonlinear Dynamics, Newcastle upon Tyne, UK).

**Table 1:** Primary antibodies used in Western blotting.

Antibody	company	Catalog Number
α-tubulin	Sigma	T 6199
Cleaved caspase-3	BioVision	3015–100
PARP-1	Santa Cruz	H-250
LC3B	Gene Tex	GTX127375
eIF2α	Gene Tex	GTX101241
p-eIF2α	Gene Tex	GTX61039
ATF4	Gene Tex	GTX89973
BiP/GRP78	BD	610978
HO-1	Stressgen	SPA-895
Nrf2	Santa Cruz	sc-722
Lamin A/C	Gene Tex	GTX101127
ERK	Cell Signaling	4695
p-ERK	Cell Signaling	4370
p38	Cell Signaling	9212
p-p38	Cell Signaling	9215
JNK	Cell Signaling	9258
p-JNK	Cell Signaling	4668

### Intracellular ROS Analysis

Flow cytometry was used to analyze intracellular reactive oxygen species with the fluorescence probe 2',7'-dichlorodihydrofluorescein diacetate (H_2_DCFDA) (Invitrogen), which passively diffuses into the cell and is cleaved and oxidized to 2',7'-dichlorofluorescein. PC12 cells (1×10^6^/ml) were seeded on 6-well plates in serum-free RPMI-1640 medium and then treated with test flavone or with an equivalent volume of DMSO vehicle (final concentration of 0.1%) for 15 min, followed by being loaded with 5 μM H_2_DCFDA for 15 min and then 25 μM 4-HNE or 100 μM H_2_O_2_ for additional 15 min. Cells were then washed twice with cold PBS and analyzed immediately [37]. Three independent samples of 10,000 cells were analyzed for each experimental condition, and the mean fluorescence intensity (MFI) was obtained.

### RNA Extraction, Real Time RT-PCR, and Semi-Quantitative RT-PCR

Total cellular RNA was prepared using Illustra RNAspin Mini RNA Isolation Kit (GE Healthcare, Buckinghamshire, UK). RT was carried out using 1 μg RNA and High-Capacity cDNA Archive Kit (Life Technologies). Real-time PCR was performed with 2 μl of the cDNA obtained above in 25 μl containing 200 nM primers **(Table 2**) and Power SYBR Green PCR Master Mix (Life Technologies). Amplification was conducted in an ABI StepOne Real-Time PCR System. The PCR conditions were as follows: 95°C for 2 min, 40 cycles at 94°C for 15 s, and 60°C for 60 s. Target gene expression was measured and normalized to the respective β-actin expression level. The identity and purity of the amplified product was checked through analysis of the melting curve carried out at the end of amplification. Relative expression was evaluated with the ΔΔCT method.

**Table 2:** Primer pairs used in RT-Q-PCR.

Gene	Primer Sequence (5'-3')	Amplicon
β-actin [81]	(F) CCTCTGAACCCTAAGGCCAA	90
	(R) AGCCTGGATGGCTACGTACA	
ATF4 [82]	(F) CTTCTCCAGGTGTTCCTCGT	163
	(R) TGCTCAGCCCTCTTCTTCTG	
CHOP	(F) AAGAATCAAAAACCTTCACTACTCTTGACC	91
	(R) TGGGAGGTGCTTGTGACCTCTGC	
GADD34	(F) TGCTCGACGCATTGCCCAGG	82
	(R) AAGGCGTGTCCATGCTCTGGC	
GCLC [83]	(F) TGGCCAGCCGTACGGAGGAA	143
	(R) CAGGGAGCCTAGCCTGGGA	
GRP78	(F) CAACTCACGTCCAACCCGGAGAA	171
	(R) TGTCTTGGTTTGCCCACCTCCG	
HO-1 [37]	(F) GCCTGCTAGCCTGGTTCAAG	87
	(R) AGCGGTGTCTGGGATGAACTA	
Nrf2 [83]	(F) GAGACGGCCATGACTGAT	196
	(R) GTGAGGGGATCGATGAGTAA	
TRB3 [82]	(F) GGACAAGATGCGAGCCACAT	179
	(R) CCACAGCAGGTGACAAGTCT	
xCT	(F) GACAGTGTGTGCATCCCCTT	110
	(R) GCATGCATTTCTTGCACAGTTC	

For the mRNA levels of XBP1s and XBP1u, the cDNA product obtained above was subjected to 30 cycles of PCR in a thermocycler (TPersonal, Biometra) using the forward primer TTACGAGAGAAAACTCATGGGC [38] and reverse primer GCGTCAGAATCCATGGGA [12] specific for rat XBP1. The following PCR conditions were used: 95°C for 5 min and 30 cycles at 95°C for 1 min, 55°C for 1 min, and 72°C for 1 min, followed by 5% polyacrylamide electrophoresis and ethidium bromide staining. The intensities of these bands were analyzed with Phoretix Gel Analysis Software (Nonlinear Dynamics, Newcastle upon Tyne, UK).

### Statistical Analysis

All experiments were repeated at least three times. The results were analyzed using one-way ANOVA with the post-hoc Tukey test by SPSS version 18, and a *p* value of < 0.05 was taken to be significant.

## Results

### Effects of Luteolin, Apigenin and Chrysin on 4-HNE-Mediated Cell Death, Caspase-3 Activation and PARP-1 Cleavage in PC12 Cells

4-HNE at high levels promotes the formation of adducts and apoptosis or necrosis [39]. We reported earlier that 4-HNE caused PC12 cell death in a dose- and time-dependent manner [12]. In this research three structurally related flavones, luteolin, apigenin and chrysin (**Fig 1A**), were used to evaluate their protective effects against 4-HNE-induced cytotoxicity. **Fig 1B** shows that treatment of PC12 cells with luteolin and apigenin (10 and 20 μM) significantly attenuated cell death caused by 25 μM 4-HNE, while chrysin did not have any protective effect, as measured by MTT assay. Luteolin (10 and 20 μM) and apigenin (20 μM) also significantly protected PC12 cells from toxicity caused by 50 μM 4-HNE; while 10 μM apigenin and chrysin (10 and 20 μM) could not. The cytoprotective effects of flavones were reconfirmed by Calcein AM staining as described in the Materials and Methods section (data not shown).

Previously, we have found that 4-HNE induced caspase-3 activation in PC12 cells [12]. Caspase-3 is considered to be the most important of the executioner caspases and ultimately causes the morphological and biochemical changes seen in apoptotic cells [40]. Poly (ADP-ribose) polymerase-1 (PARP-1) is one of several known cellular substrates of caspases and cleavage of PARP-1 by caspases is considered to be a hallmark of apoptosis [41]. **Fig 1C** shows that treatment of PC12 cells with 4-HNE (25 μM) for 6 h induced a marked level of caspase-3 activation and PARP-1 cleavage. This result supports at least in part the notion that in neural cells, 4-HNE induces apoptosis through caspase-3 activation [42]. Luteolin (20 μM) significantly inhibited 4-HNE-mediated activation of caspase-3 and PARP-1. On the other hand, apigenin (20 μM) did not significantly change caspase-3 activation but decreased PARP-1 cleavage markedly; indicating 4-HNE-mediated activation of other caspases may be attenuated. In comparison, chrysin (20 μM) increased 4-HNE-induced caspase-3 and PARP-1 cleavage.

### Effects of Luteolin, Apigenin and Chrysin on 4-HNE-Mediated LC3 Conversion and Intracellular ROS Overproduction in PC12 Cells

**Fig 2:**
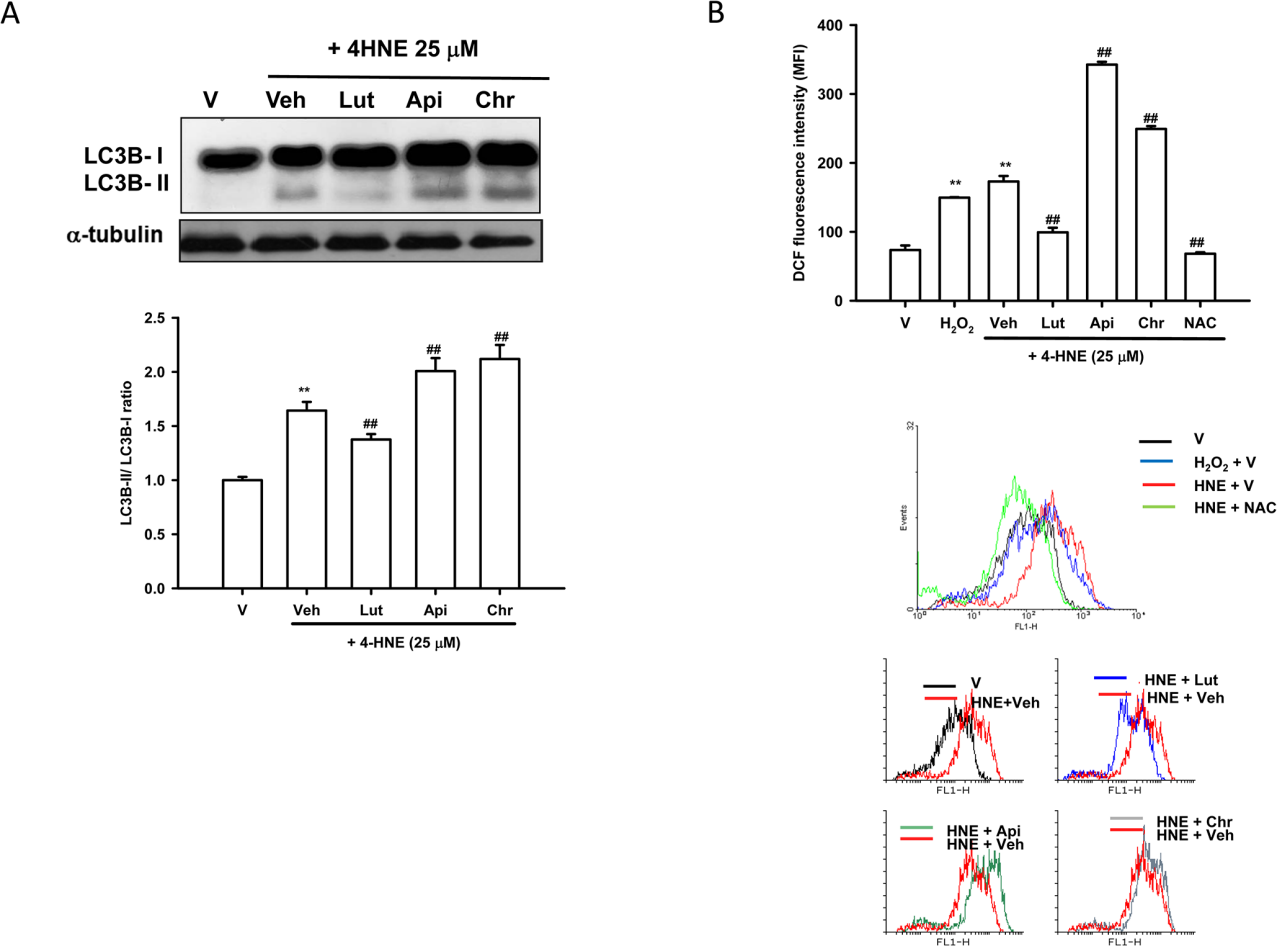
Effects of luteolin, apigenin and chrysin on 4-HNE-mediated LC3 conversion and ROS overproduction in PC12 cells. (**A**) Western blot analysis of conversion of LC3B. PC12 cells were treated with luteolin, apigenin and chrysin (20 μM) 30 min prior to 4-HNE (25 μM) treatment in serum-free medium for 6 h at 37°. Cell lysates were prepared and were subjected to SDS-PAGE. Immunoblotting was then carried out using an anti-LC3B or anti-α-tubulin antibody. The blots are representative from one of three independent experiments. Data obtained from immunoblots were then analyzed using Phoretix Gel Analysis Software as described under Materials and methods. (**B**) Intracellular ROS production was measured by DCFDA as described in Materials and Methods. Data represent the mean ± SD of three independent experiments. **, *p*<0.01 represents significant differences compared with vehicle control (without oxidant). ##, *p*<0.01 represents significant differences compared with the 4-HNE-treated vehicle group.

It has been reported that exposure to 4-HNE led to a concentration-dependent increase in LC3 (rat microtubule-associated protein 1 light chain 3)-II formation in vascular smooth-muscle cells (VSMCs) [43]. LC3 is required for the formation of autophagosome membranes and the conversion of LC3 from LC3-I (free form, 18 kDa) to LC3-II (phosphatidylethanolamine-conjugated form, 16 kDa) is an initiating step in autophagy in mammals. The abundance of LC3-II correlates with the number of autophagosomes and is therefore a practical index of autophagic activity in mammalian cells [44]. **Fig 2A** shows an increase in the LC3B-II 6 h after 4-HNE (25 μM) treatment in PC12 cells. Co-treatment of PC12 cells with 20 μM luteolin significantly counteracted LC3B-II accumulation; however, 20 μM apigenin and chrysin enhanced 4-HNE-mediated LC3B-II levels.

It has been reported that macroautophagy stimulator rapamycin induced LC3-II accumulation in PC12 cells [45]. We found that rapamycin treatment effectively caused PC12 cell death dose-dependently and the combination of rapamycin and 4-HNE enhanced cytotoxicity **(S1 Fig).** This result indicates that the activation of the autophagic machinery may exacerbate 4-HNE-induced PC12 cell damage. Furthermore, the addition of luteolin and apigenin (10 and 20 μM) effectively blocked rapamycin-induced cytotoxicity (**S2 Fig**).

We reported previously that 4-HNE dose-dependently induced ROS overproduction [12]. Although luteolin and apigenin are generally believed to be antioxidants, they are found to induce ROS accumulation [46,47]. We thus studied whether luteolin or apigenin could attenuate 4-HNE-stimulated PC12 cell death through reducing intracellular ROS production. **Fig 2B** shows that treatment of PC12 cells with 4-HNE (25 μM) or H_2_O_2_ (100 μM), positive control, stimulated intracellular ROS overproduction promptly. Treatment of cells with a well-known antioxidant, NAC (1 mM), completely abrogated 4-HNE-mediated ROS production in PC12 cells. Similarly, luteolin (20 μM) prevented the 4-HNE-mediated ROS overproduction. However, apigenin and chrysin (20 μM) markedly enhanced the ROS overproduction. This result indicates that the cytoprotective effect of luteolin, but not apigenin, may be associated with a decrease in intracellular ROS.

### Luteolin and Apigenin Enhance 4-HNE-Activated XBP-1 Splicing and Translation of ATF4 and GRP78

**Fig 3:**
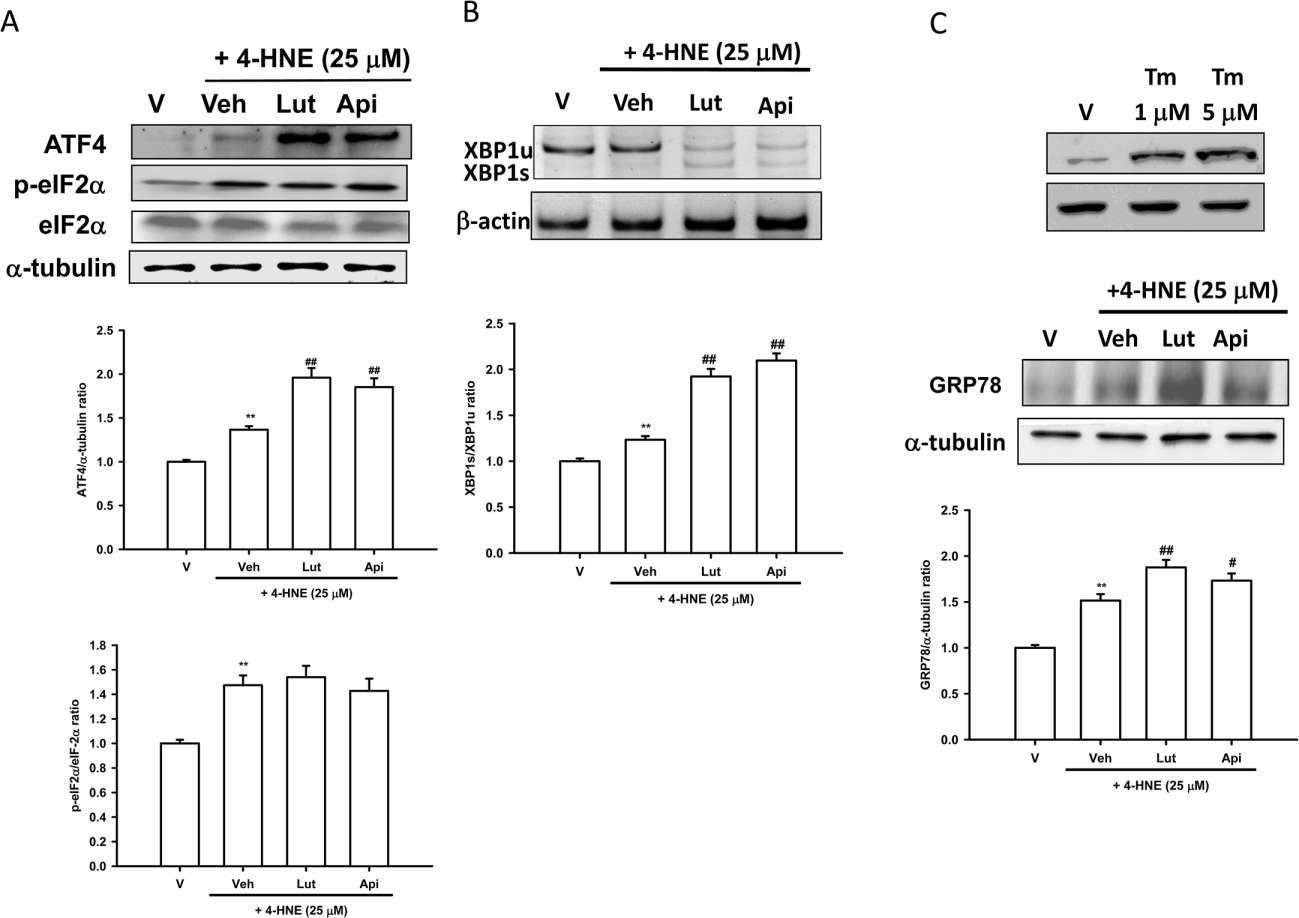
Luteolin and apigenin enhance 4-HNE-mediated unfolded protein response. (**A**) Western blot analysis of ATF4 and eIF2α phosphorylation in lysates obtained from PC12 cells incubated in the presence of vehicle only (V), or presence of 25 μM HNE plus vehicle (Veh), or co-treatment with 20 μM luteolin or apigenin for 2 h. (**B**) Effects of flavones on XBP1 splicing. PC12 cells were treated with luteolin and apigenin (20 μM) 30 min prior to 4-HNE (25 μM) treatment for 4 h at 37°. RNA was prepared and semi-quantitative RT-PCR was used for the analysis of mRNA levels of XBP1s and XBP1u, as described in Materials and Methods. (**C**) Western blot analysis of GRP78 in cell lysates prepared from PC12 cells, which were pretreated with luteolin or apigenin (20 μM) for 30 min followed by 4-HNE for 6 h. Immunoblots and polyacrylamide gels were then analyzed using Phoretix Gel Analysis Software as described under Materials and methods. Data represent the mean ± SD of three independent experiments. **, *p*<0.01 represents significant differences compared with vehicle control (without oxidant). ##, *p*<0.01 represents significant differences compared with the 4-HNE-treated vehicle group.

We reported previously that 4-HNE triggers ER stress, activates three canonical arms of the unfolded protein responses, and induces pro-apoptotic CHOP expression in PC12 cells [12]. To investigate how luteolin and apigenin affect 4-HNE-mediated UPR in PC12 cells, we started with analyzing PERK-eIF2α pathway, which is thought to be the most important stress response pathway leading to attenuation of global protein translation. Some selected proteins with internal ribosomal entry sites (IRES), such as ATF4 and GRP78/BiP, are translated more efficiently [48]. **Fig 3A** shows that PC12 cells treated with 25 μM 4-HNE for 2 h led to increases in eIF2α phosphorylation and ATF4 protein expression. Co-treatment of PC12 cells with 4-HNE (25 μM) and luteolin or apigenin (20 μM) did not change 4-HNE-mediated eIF2α phosphorylation, but significantly enhanced ATF4 protein expression.

The transcriptional factor X-box-binding protein 1 (XBP1) is activated by accumulating unfolded proteins and other endoplasmic reticulum (ER) stress factors, and protects cells from oxidative stress [49]. PC12 cells were pre-treated with flavones for 30 min before 4-HNE (25 μM) exposure for 4 h. The levels of the unspliced (XBP1u) and active spliced XBP1 (XBP1s) mRNA were measured by RT-PCR and polyacrylamide electrophoresis, as described in Materials and Methods. In accordance with previous finding [12], 4-HNE (25 μM) slightly increased the level of XBP1s as compared with vehicle control. Co-treatment of cells with 4-HNE and luteolin or apigenin (20 μM) significantly enhanced XBP1 splicing (**Fig 3B**).

GRP78/BiP is a central regulator for ER stress due to its role as a major ER chaperone with anti-apoptotic properties. Western blotting (**Fig 3C**) revealed that treatment of PC12 cells with a common ER stress inducer, tunicamycin (Tm, 1 μM), for 6 h significantly induced GRP78 protein expression. In comparison, 25 μM 4-HNE caused a weaker induction. Co-treatment of PC12 cells with 25 μM 4-HNE and 20 μM luteolin or apigenin produced levels of GRP78 protein expression that were slightly higher than those seen when using only with 4-HNE. Overall, both luteolin and apigenin may restore ER homeostasis by enhancing 4-HNE-mediated ATF4 translation and XBP1 splicing. Luteolin upregulates 4-HNE-mediated expression of the anti-apoptotic ER chaperone GRP78 protein more significantly than apigenin does.

### Luteolin and Apigenin Down-Regulate 4-HNE-Mediated Pro-Apoptotic CHOP-Regulated Genes

**Fig 4:**
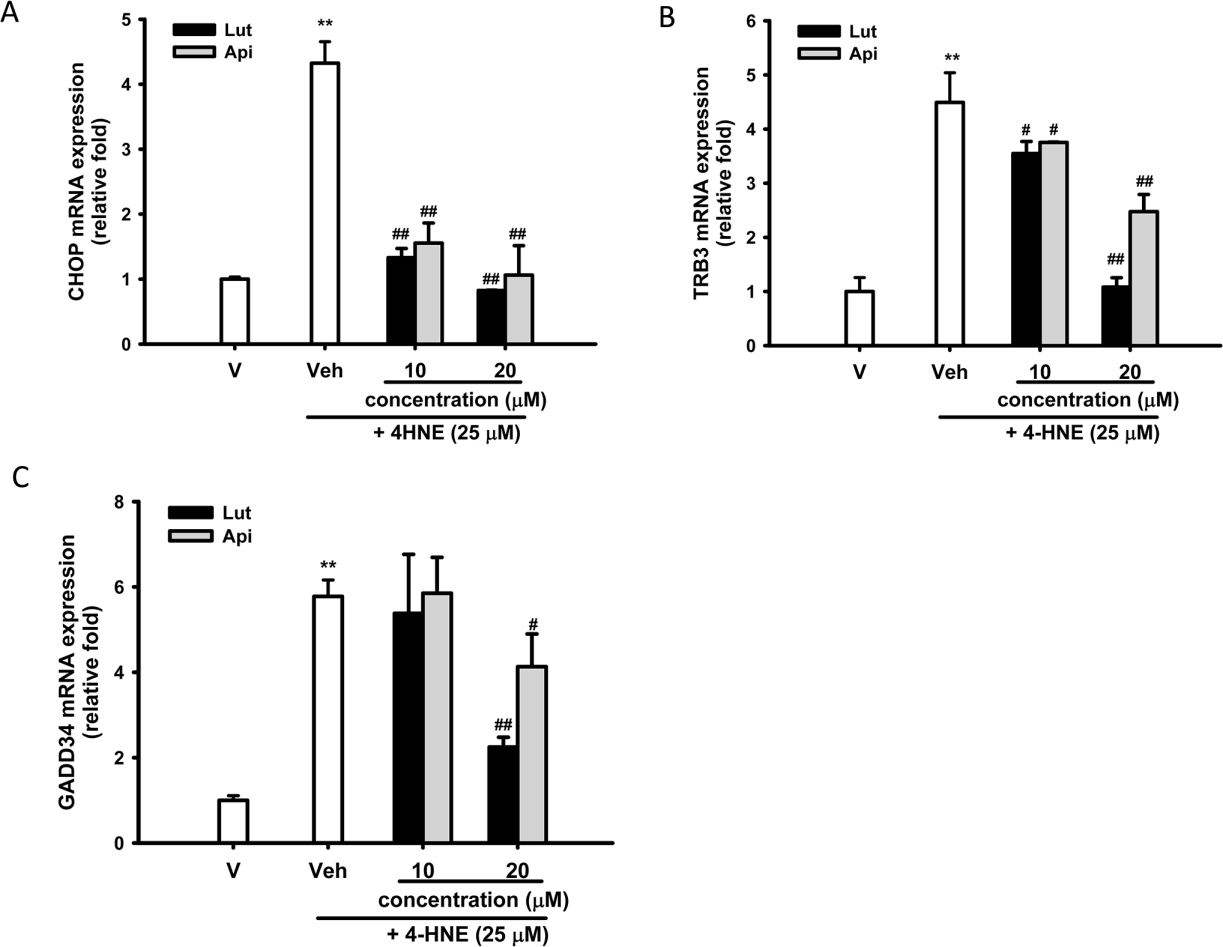
Luteolin and apigenin inhibit ER stress-induced pro-apoptotic mRNA expression in PC12 cells. PC12 cells were treated with luteolin or apigenin (20 μM) 30 min prior to 4-HNE (25 μM) treatment for 4 h at 37°. RNA was prepared and the expression of CHOP, TRB3 and GADD34 was analyzed by RT-Q-PCR and normalized to β-actin, as described in Materials and Methods. The data represent the mean ± SD of three independent experiments. **p<0.01 represents significant differences compared with vehicle control (without 4-HNE); #*p*<0.01; ##, *p*<0.01 represent significant differences compared with the 4-HNE-treated vehicle group.

Upon prolonged ER stress, UPR up-regulates transcription of CHOP, which induces transcription of pro-apoptotic genes [50]. **Fig 4A** shows that 25 μM 4-HNE induced CHOP mRNA by 4.3-fold after 4 h treatment. CHOP expression was dose-dependently attenuated by luteolin and apigenin (10 and 20 μM).

CHOP mediates expression of pro-apoptotic carbonic tribbles-related protein 3 (TRB3) [51]. **Fig 4B** shows that treatment of PC12 cells with 25 μM 4-HNE for 4 h increased TRB3 mRNA expression by 4.5-fold. Both luteolin and apigenin (10 and 20 μM) dose-dependently counteracted 4-HNE-mediated TRB3 overexpression, although luteolin was more potent in this regard.

CHOP directly activates GADD34, which promotes ER client protein biosynthesis by dephosphorylating phospho-Ser^51^ of eIF2α in stress cells [52]. **Fig 4C** shows that, in parallel to the effects on TRB3, 25 μM 4-HNE significantly upregulated GADD34 by 5.8-fold and co-treatment with 20 μM apigenin and luteolin attenuated GADD34 overexpression. In conclusion, both luteolin and apigenin attenuated the expression of ER stress-induced pro-apoptotic genes in PC12 cells, although luteolin had the greater effect.

### Effects of Luteolin and Apigenin on Nrf2-Targeted Gene Expression

**Fig 5:**
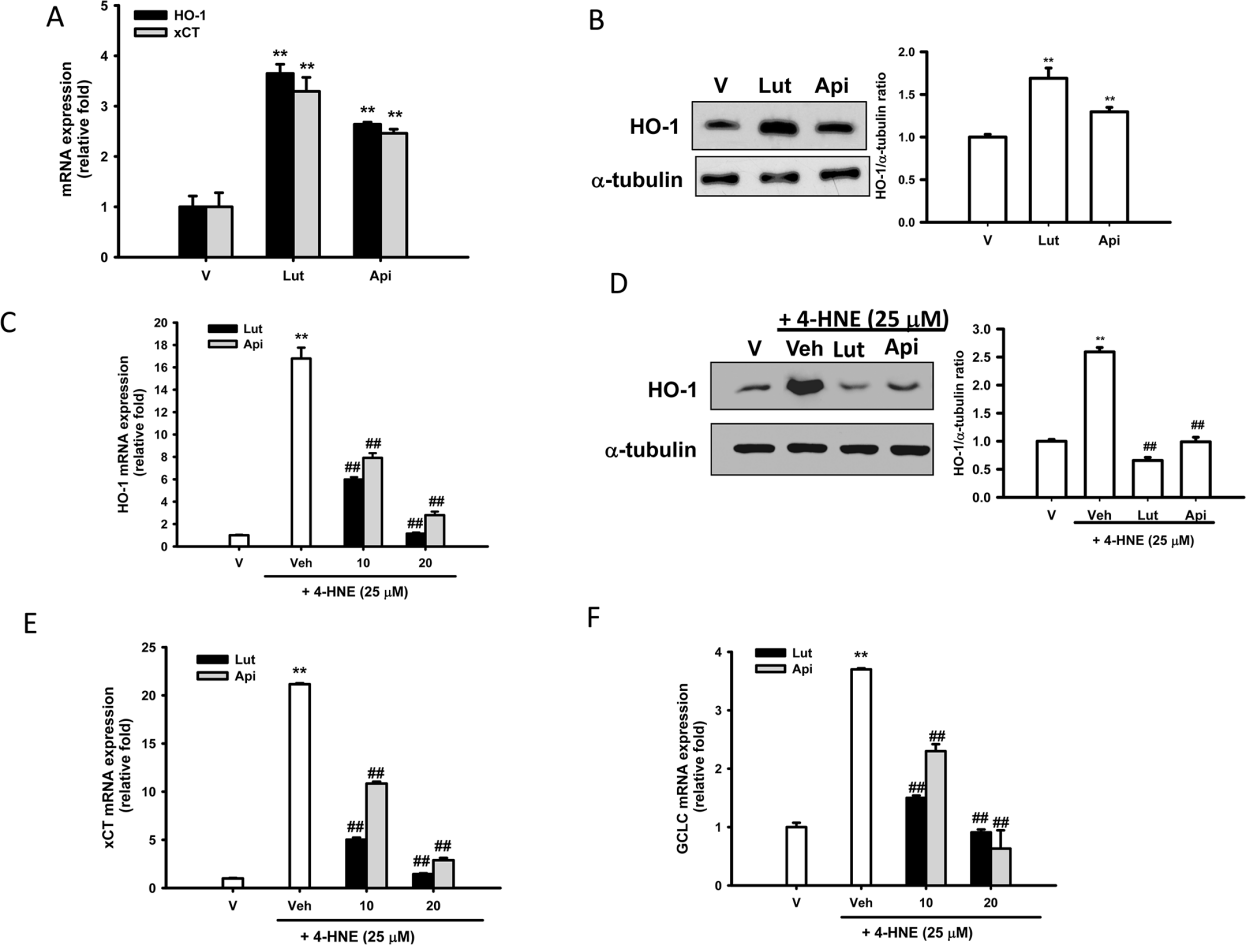
Effects of flavones on Nrf2-targeted gene expression. (**A and B**) Luteolin exerts the strongest stimulatory effects on HO-1 and xCT expression. PC12 cells were treated with 20 μM luteolin and apigenin. After 6 h, RNA was prepared and the mRNA expression of HO-1 and xCT was analyzed by RT-Q-PCR and normalized to β-actin, as described in Materials and Methods. Data represent the mean ± SD of three independent experiments. *, *p*<0.05; **, *p*<0.01 represents significant differences compared with vehicle control. After 9 h, total cell lysates were prepared and subjected to Western blotting analysis of HO-1 and α-tubulin, which was used as a loading control. (**C-F**) Luteolin and apigenin counteract 4-HNE-mediated phase II enzyme expression. PC12 cells were treated with luteolin or apigenin (10 or 20 μM) 30 min prior to 4-HNE (25 μM) treatment. After 4 h, RNA was prepared and the expression of HO-1, xCT and GCLC was analyzed by RT-Q-PCR and normalized to β-actin, as described in Materials and Methods. After 6 h, total cell lysates were prepared and subjected to Western blotting analysis of HO-1 and α-tubulin, which was used as a loading control. Data obtained from immunoblots were then analyzed using Phoretix Gel Analysis Software as described under Materials and methods. The data represent the mean ± SD of three independent experiments. **p<0.01 represents significant differences compared with vehicle control (without 4-HNE); #*p*<0.01; ##, *p*<0.01 represent significant differences compared with the 4-HNE-treated vehicle group.

Nrf2-ARE is a primary sensor and oxidative stress regulator. In addition to direct ROS scavenging, as reported shown above, luteolin prevents PC12 cells from oxidative damage by enhancing the binding of Nrf2 to ARE, and increasing the expression of HO-1 mRNA and protein [53]. **Fig 5A** shows that in the absence of 4-HNE, luteolin and apigenin (20 μM) significantly induced HO-1 and xCT mRNA expression in PC12 cells, and luteolin exerted the strongest stimulatory effect. **Fig 5B** shows that similar to our previous report [53], HO-1 protein was markedly elevated by luteolin (20 μM) treatment, and less significant effect was also noted for apigenin (20 μM) treatment.

We found earlier that 4-HNE induced Phase II enzymes through Nrf2 activation in PC12 cells [12]. **S3 Fig** shows that 4-HNE (25 μM) induced Nrf2 mRNA expression and protein translocation into nucleus. The addition of antioxidant NAC (1 mM) abolished Nrf2 mRNA overexpression and nuclear protein translocation. **Fig 5C and 5D**show that 4-HNE (25 μM) robustly induced HO-1 mRNA and protein expression. In contrast to the above-mentioned notion of being a natural Nrf2 activator, luteolin was found to be an inhibitor against xenobiotic-induced Nrf2 [54,55]. We found that co-treatment of PC12 cells with 4-HNE and luteolin or apigenin antagonized HO-1 mRNA and protein expression (**Fig 5C and 5D**). **Fig 5E** shows that a 21.2-fold increase in xCT mRNA was induced by 25 μM 4-HNE treatment for 4 h in PC12 cells. Co-treatment with luteolin and apigenin (10 and 20 μM) significantly counteracted the upregulation of xCT.

Nrf2 also regulates transcription of the catalytic subunit of GCL (glutamate-cysteine ligase), which catalyzes the first and rate-limiting step in GSH biosynthesis [56]. **Fig 5F** shows that 25 μM 4-HNE significantly increased GCLC mRNA expression by 3.7-fold. Both flavones (10 and 20 μM) significantly reduced GCLC overexpression, and luteolin and apigenin (20 μM) completely blocked the overexpression. This observation is in a good agreement with previous notion of hormetic actions of Nrf2. Modest Nrf2 activation protects cells against acute toxicity, but persistent activation of Nrf2 causes more harm than benefit [57,58].

### Luteolin and Apigenin Activate Mitogen-Activated Protein Kinase (MAPK) Signaling Pathways

**Fig 6:**
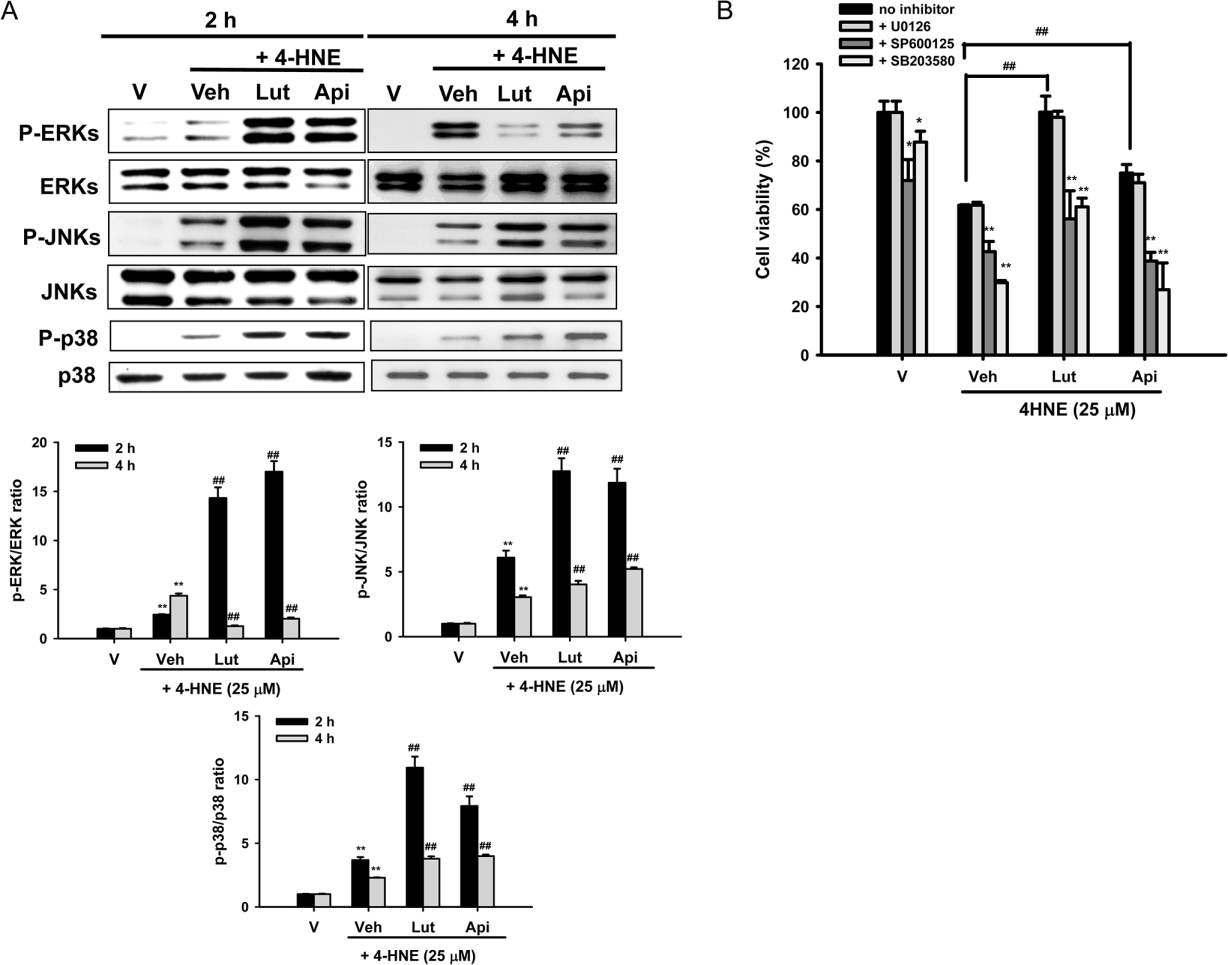
Contributions of MAPK signaling pathways to the cytoprotective effects of luteolin and apigenin. (**A**) Effects of flavones on MAPK activation. PC12 cells were treated with luteolin or apigenin (20 μM) 30 min prior to 4-HNE (25 μM) treatment for 2 and 4 h at 37°. Cell lysates were prepared and the levels of MAPK activation were analyzed by Western blotting. Immunoblots were then analyzed using Phoretix Gel Analysis Software as described under Materials and methods. Data represent the mean ± SD of three independent experiments. **, *p*<0.01 represents significant differences compared with vehicle control (without oxidant). ##, *p*<0.01 represents significant differences compared with the 4-HNE-treated vehicle group. (**B**) Effects of inhibition of MAPK activation on cell viability. PC12 cells were pretreated for 30 min with 5 μM ERK (U0126), JNK (SP600125) or p38 MAPK inhibitor (SB203580) and 20 μM luteolin or apigenin was then added 30 min prior to 4-HNE (25 μM) exposure for 16 h. MTT was used to analyze the cell viability. Data represent the mean ± SD of three independent experiments. **p<0.01 represents significant differences compared with respective no inhibitor group. ##, *p*<0.01 represents significant differences compared with the 4-HNE-treated vehicle group. Data represent the mean ± SD of three independent experiments.

Mitogen-activated protein kinases compose a family of protein kinases that play an essential role in relaying extracellular signals from the cell membrane to the nucleus via a cascade of phosphorylation events [59]. It was found that 4-HNE treatment for 15 min to 2 h induced activation of extracellular signal-regulated kinase (ERK), NH_2_-terminal kinase (JNK), and p38 MAPK [12]. To investigate how flavones affect 4-HNE-mediated MAPK pathways, PC12 cells were treated with 20 μM luteolin or apigenin for 30 min prior to 4-HNE (25 μM) exposure for 2 or 4 h, and cell lysates were immunoblotted with phospho-specific antibodies, as described in the Materials and Methods. **Fig 6A** shows that all three MAPK pathways were activated 2 h and 4 h after 4-HNE exposure. Co-treatment of cells with 4-HNE and luteolin or apigenin (20 μM) for 2 h significantly augmented all three MAPK pathways. The enhancement in JNK and p38 activation by luteolin and apigenin decreased slightly after 4 h. 4-HNE treatment for 4 h exerted a stronger induction effect on ERK phosphorylation than 2 h treatment, but neither of the tested flavones enhanced ERK activation after 4 h treatment. Similar trend was observed for 10 μM luteolin and apigenin (data not shown).

To assess which of the MAPK pathways are involved in the cytoprotective effects of luteolin and apigenin, PC12 cells were pre-incubated for 30 min with 5 μM inhibitor for each pathway, namely U0126 (ERK), SP600125 (JNK) and SB203580 (p38 MAPK), and 20 μM luteolin or apigenin were then added 30 min prior to 4-HNE stimulation for 16 h. **Fig 6B** shows that addition of JNK inhibitor SP600125 and p38 MAPK specific inhibitor SB203580, but not MEK inhibitor U0126, significantly exacerbated 4-HNE-mediated cytotoxicity. These results confirm that the JNK and p38 MAPK signaling pathways may be associated with 4-HNE-mediated stress responses [12]. Furthermore, addition of SP600125 and SB203580 also significantly attenuated the cytoprotective effects of luteolin and apigenin, suggesting the possible involvement of JNK and p38 MAPK in their cytoprotective effects.

### Effects of JNK and p38 MAPK Inhibitors on CHOP, HO-1 and xCT Transcription

**Fig 7:**
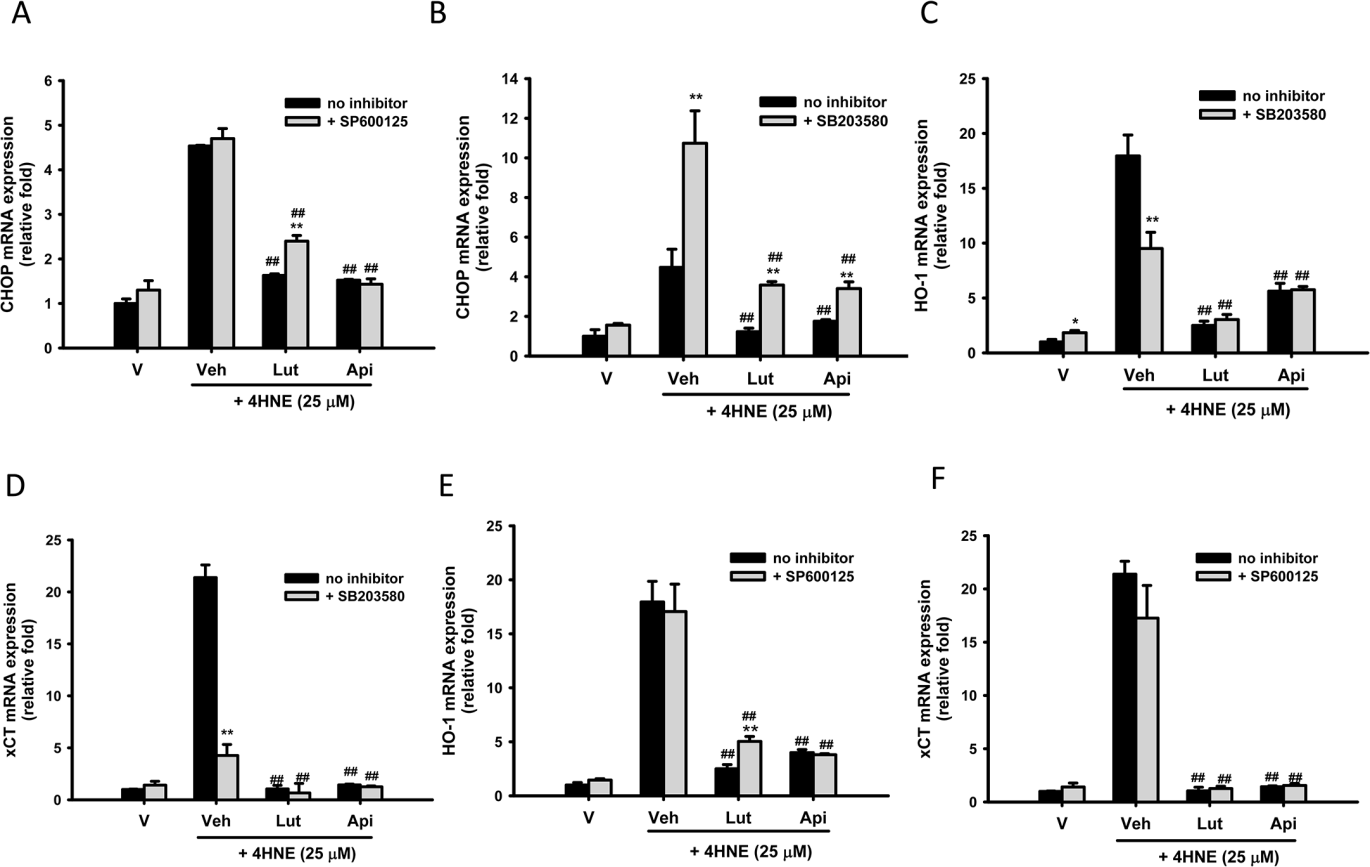
Effects of JNK and p38 MAPK inhibitors on CHOP, HO-1 and xCT expression in response to 4-HNE. PC12 cells were pretreated for 30 min with 5 μM JNK (SP600125) or p38 MAPK inhibitor (SB203580) and 20 μM luteolin or apigenin was then added 30 min prior to 4-HNE (25 μM) exposure for 4 h. The mRNA expression of CHOP, HO-1 and xCT was analyzed by RT-Q-PCR and normalized to β-actin, as described in Materials and Methods. Data represent the mean ± SD of three independent experiments. **p<0.01 represents significant differences compared with respective no inhibitor group. ##, *p*<0.01 represents significant differences compared with the 4-HNE-treated vehicle group.

We found previously that p38 MAPK activation may serve as an upstream negative regulator for ER stress-mediated proapoptotic gene expression [12]. Following the same line, **Fig 7A and 7B**show that addition of SB203580 (5 μM), but not SP600125, increased 4-HNE-induced CHOP, highlighting that the p38 MAPK signaling pathway, instead of JNK, has a negative effect on pro-apoptotic gene expression. Furthermore, the attenuation of 4-HNE-mediated CHOP expression by luteolin was significantly counteracted by both SB203580 and SP600125 (5 μM). On the other hand, apigenin-mediated CHOP inhibition was only antagonized by SB203580, but not SP600125. Neither SB203580 nor SP600125 affected anti-apoptotic ER marker GRP78 protein expression in PC12 cells (data not shown). These results indicate that luteolin and apigenin serve as p38 MAPK activators, which can attenuate CHOP overexpression.

We reported previously that addition of p38 MAPK specific inhibitor SB203580 attenuated 4-HNE-mediated HO-1 upregulation [12]. In accordance with this, **Fig 7C and 7D**show that SB203580 treatment significantly decreased 4-HNE-induced HO-1 and xCT overexpression. This result is in agreement with the notion that p38 MAPK signals survival under moderate oxidative stress through up-regulation of antioxidant defenses [19]. However, the inhibitory activities of luteolin and apigenin on Phase II enzyme expression were not affected by SB203580. **Fig 7E and 7F**demonstrate that 4-HNE-upregulated HO-1 and xCT expression was not affected by JNK inhibitor SP600125. However, luteolin-mediated attenuation of HO-1 overexpression was counteracted by SP600125 (5 μM). These results suggest that luteolin-activated JNK signaling may be involved in down-regulation of stress-induced HO-1 expression.

## Discussion

4-HNE serves as a marker of oxidative stress and a causative agent for cancers, metabolic syndrome and neurodegenerative diseases [60]. This study found that 4-HNE caused cell death at least in part through inducing apoptosis and autophagy in PC12 cells, as indicated by the increases in active caspase-3, PARP-1 cleavage and LC3 conversion. Among three tested flavones, luteolin exhibited the strongest anti-apoptotic effect, followed by apigenin, while chrysin did not have any protective effect. Luteolin also decreased LC3-II accumulation, indicating autophagy might also been attenuated. In contrast, apigenin and chrysin enhanced 4-HNE-mediated LC3 conversion. There are inconsistent reports regarding the effects of luteolin and apigenin on apoptosis and autophagy. Both luteolin and apigenin have been shown as apoptosis and autophagy inducers, which may support their possible chemopreventive or neuroprotective roles [30,61,62]. On the other hand, it was reported that they inhibited apoptosis or autophagy [27,62]. In this report, we found that luteolin and apigenin (10 and 20 μM) significantly inhibited autophagy stimulator rapamycin-mediated cytotoxicity in PC12 cells, indicating they might work as autophagy inhibitors at current concentrations.

It is known that the ability of 4-HNE to form adducts with proteins is the major deleterious event it promotes, and the extent of apoptosis is directly dependent on the yield of 4-HNE-adducts that are accumulated in the cells. We found that levels of 4-HNE-protein adducts, measured 15 min to 4 h after exposure to 4-HNE (25 μM), were not significantly affected by any of the tested flavones (data not shown), indicating the protective effects of luteolin and apigenin did not result from direct 4-HNE scavenging. This result is in a good agreement with a previous report which shows that flavones are not a typical type of 4-HNE scavengers [63].

Luteolin and apigenin are regarded as both ROS scavengers and generators [46,47]. We found that co-treatment with luteolin blocked 4-HNE-induced intracellular ROS, but co-treatment with apigenin and chrysin sensitized toward ROS formation.

It has been reported that ER is a direct target of 4-HNE, and that UPR is activated in 4-HNE-treated cells [9–12]. Because XBP1 splicing occurs exclusively by IRE1 activation, the formation of the faint band of XBP1s indicates that 4-HNE only slightly activated the IRE1 pathway. In addition, luteolin and apigenin further enhanced 4-HNE-mediated ATF4 translation. Under ER stress, cells activate GRP78 (also known as BiP), which protects them from lethal conditions, and CHOP (also known as GADD153), which plays major roles in ER stress-induced apoptosis [64,65]. We observed that 4-HNE induced the expression of GRP78 and CHOP in PC12 cells. CHOP-induced pro-apoptotic TRB3 and GADD34 were also upregulated by 4-HNE. Co-treatment of cells with 4-HNE and luteolin or apigenin further stimulated GRP78 expression and simultaneously attenuated pro-apoptotic genes, CHOP, TRB3 and GADD34 expression, and the effect of luteolin was more profound than that of apigenin.

Tunicamycin (Tm) induces ER stress by inhibiting the glycosylation of newly synthesized proteins and also leads to ER stress-mediated apoptosis via an intracellular mechanism activating the caspase cascade. It has been reported that luteolin significantly suppressed Tm-induced caspase-3 activation without changing Tm-induced CHOP or GRP78 expression in SH-SY5Y neuroblastoma cells [66]. We thus investigated how luteolin and apigenin affect Tm-induced cytotoxicity and ER stress gene expression in PC12 cells. **S4A Fig** shows that Tm (1 μM) significantly induced PC12 cell death, and cotreatment with luteolin and apigenin (20 μM) completely blocked cytotoxicity as measured by MTT and reconfirmed by Calcein AM staining.

Tm (1 μM) strongly induced XBP1 mRNA processing and GRP78 expression (**S4B and S4C Fig**). In contrast to the above mentioned notion being inducers for 4-HNE-mediated XBP1 splicing and GRP78 expression, luteolin and apigenin (10 and 20 μM) attenuated Tm-mediated XBP1 processing and GRP78 induction in dose-dependent manners. Nevertheless, luteolin and apigenin inhibited Tm-induced expression of pro-apoptotic genes, CHOP, TRB3 and GADD34 in PC12 cells **(S4D, S4E and S4F Fig)**. These results indicate that although the cytotoxicity caused by 4-HNE and Tm can be effectively attenuated by luteolin and apigenin, the underlying inhibitory mechanisms against these two ER stress stimulators are distinct.

Exposure of cells to 4-HNE results in the modification of Keap1 at several important cysteine residues, and stabilization and release of Nrf2 to the nucleus [67]. Previously we showed that 4-HNE-induced HO-1 and GCLC expression is mediated through Nrf2 activation [12]. We showed here again that 4-HNE induced Nrf2 expression and nuclear translocation and a dramatic rise in the target genes, HO-1 and xCT, and modest increase in GCLC in PC12 cells. A growing body of evidence shows the hormetic actions of HO-1 and xCT. Sustained HO-1 over-expression contributes to the iron sequestration, intracellular oxidative stress and mitochondrial damage documented in aging-related neurodegenerative disorders, such as Alzheimer’s disease and PD [68,69]. xCT is the transporter subunit of the heterodimeric amino-acid transporter system Xc^-^, which functions as an exchange transporter for cystine/glutamate, catalyzing the entry of cystine and the exit of glutamate [60]. However, there are more reports in the literature describing the role of glutamate released by system Xc^-^ in brain disease than about protective effects of increased cystine availability, and thus GSH synthesis [70]. Therefore, the high level expression of Nrf2-targeted genes reflects the intense stress induced by 4-HNE, and this may be harmful rather than protective for PC12 cells.

Polyphenolic compounds, especially those containing catechol and hydroquinones, exert an ARE-induction effect after oxidation to their corresponding electrophilic quinones that contain Michael acceptors [13]. Following this line, we previously reported that luteolin is a mild ARE inducer and induces moderate HO-1 expression, so as to exert cytoprotective effects in PC12 cells [53]. Luteolin and apigenin also activate Nrf2-ARE in rat primary hepatocytes [71], HepG2 cells [72] and *in vivo* [73]. However, luteolin was found to inhibit expression of Nrf2 and Phase II enzymes in response to 2,3,7,8-tetrachlorodibenzop-dioxin (TCDD) or tert-butylhydroquinone (tBHQ) in HepG2 cells [74]. We also found the luteolin inhibited 6-OHDA-mediated Phase II enzyme expression in PC12 cells [29]. In the current work we found that in the absence of 4-HNE, luteolin stimulated HO-1 and xCT expression more strongly than apigenin did, supporting the importance of the catechol structure in ARE induction [13]. On the other hand, luteolin and apigenin inhibited 4-HNE-mediated HO-1, xCT, and GCLC mRNA expression and luteolin exerted the stronger inhibitory effect. Based on the previous and current results, these flavones can serve as activators or inhibitors of Nrf2 depending on the presence or absence of electrophilic xenobiotics. In the presence of 4-HNE, luteolin can serve as both ROS scavenger and Nrf2 inhibitor; while apigenin serves only as Nrf2 inhibitor, but not ROS scavenger. The inhibitory effects of flavones against 4-HNE-mediated Nrf2-targeted gene expression had the same trend as their stimulatory effects, indicating that the preconditioning effects induced by flavones confer an adaptive response to 4-HNE-induced Phase II enzyme expression and cytotoxicity.

We also found that luteolin and apigenin enhanced 4-HNE-activated ERK, JNK and p38 MAPK, and attenuation of JNK and p38 MAPK phosphorylation by the addition of specific inhibitors counteracted their cytoprotective effects. Inhibition of luteolin- and apigein-activated p38 MAPK also augmented ER stress-induced apoptotic CHOP. The above results suggest that luteolin- and apigenin-enhanced p38 MAPK activation may confer an adaptive response to resist 4-HNE-mediated ER stress and cytotoxicity.

Numerous studies have demonstrated that the upregulation of HO-1 requires p38 MAPK activation [12,75–77]. In agreement with previous findings, we found that SB203580 (p38 inhibitor), but not PD098059 (JNK inhibitor), attenuated 4-HNE-mediated HO-1 and xCT expression, supporting the view that 4-HNE induces Nrf2-targeted gene expression via sequential activation of the p38 MAPK in PC12 cells [12]. It was also recently reported that p38 MAPK mediates cell survival in response to oxidative stress via induction of other antioxidant enzymes, such as SOD and catalase [19].

Protein kinase A (PKA), a canonical signal transducer of cAMP, plays pivotal roles in neuronal outgrowth, survival and regeneration. It was reported previously that 4-HNE stimulates PKA activation in adipocytes [78] and the cytoprotective effect of luteolin is related to elevated PKA activity [79]. **S5A Fig** shows that H89, a potent PKA inhibitor, significantly alleviated 4-HNE-mediated cytotoxicity and limited the cytoprotective effects of luteolin and apigenin, indicating PKA signaling may be protective in response to 4-HNE. **S5B Fig** shows that CHOP expression in both vehicle- and 4-HNE-treated cells was significantly enhanced by H89, but not in flavone-co-treated cells. These data suggest that PKA signaling may be involved in maintaining ER homeostasis in response to 4-HNE, because inhibition of PKA causes upregulation of CHOP. **S5C and S5D Fig**show that 4-HNE-mediated expression of HO-1, and xCT were both significantly enhanced by H89, indicating PKA serve as a negative regulator for Nrf2-mediated oxidative stress related gene expression. However, the inhibitory effects of luteolin and apigenin on 4-HNE-mediated expression of HO-1 or xCT were not affected by H89. Furthermore, effects of H89 that are unrelated to its inhibition of PKA have been observed [80]. Therefore more experiments are needed to clarify the role of PKA in the cytoprotective effects of luteolin and apigenin.

**Fig 8:**
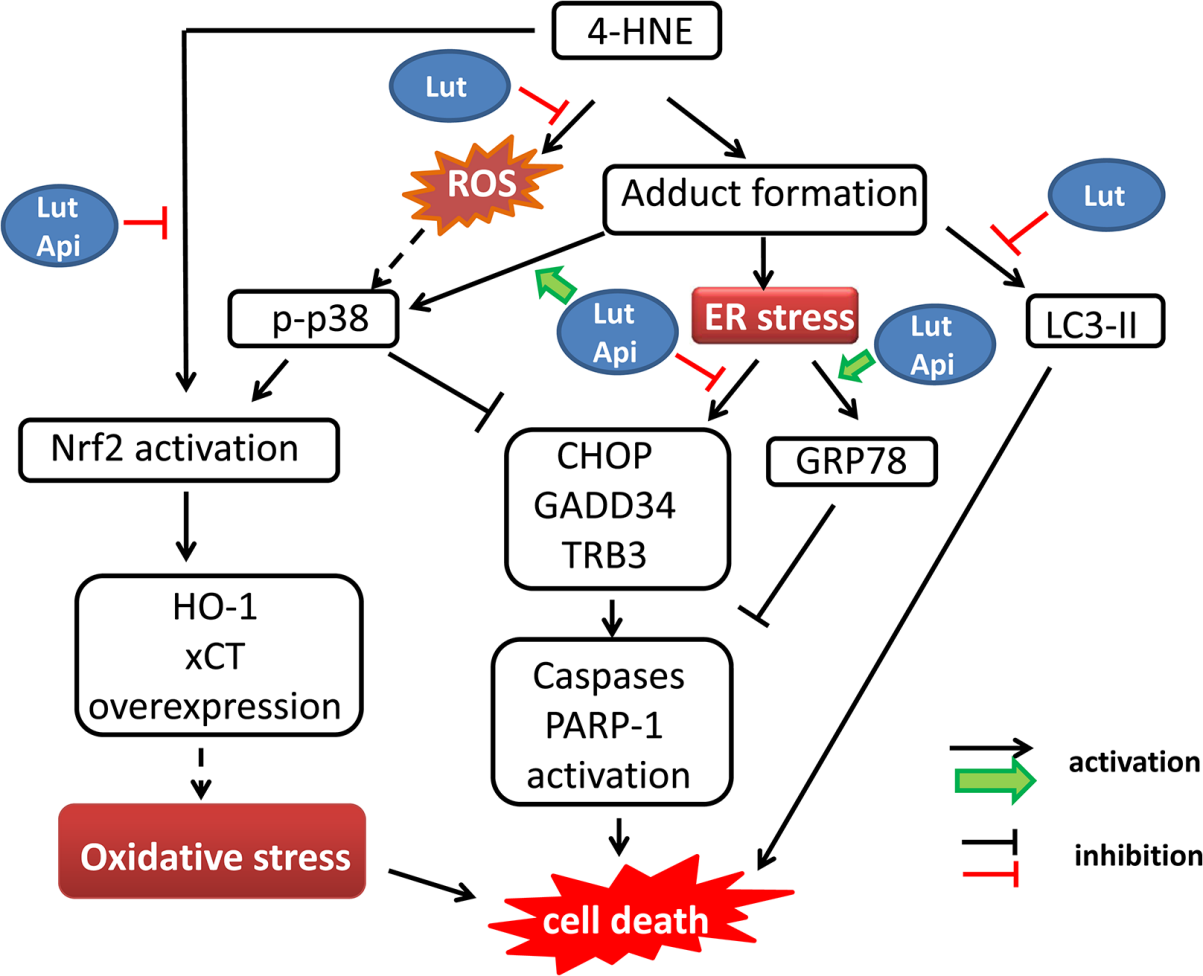
Hypothetic mechanism of luteolin and apigenin in preventing 4-HNE-mediated cell death in PC12 cells. Luteolin can inhibit 4-HNE-induced intracellular ROS over-production and LC3-II accumulation. Luteolin and apigenin modulate 4-HNE-mediated unfolded protein response (UPR), leading to an increase in endoplasmic reticulum chaperone GRP78 and decrease in the expression of UPR-targeted pro-apoptotic genes. They also induce p38 MAPK activation and counteract the expression of Nrf2-targeted HO-1 and xCT in the presence of 4-HNE. The activation of caspase-3 and PARP-1 are attenuated and cell viability is restored. The dashed lines indicate hypothesis not tested yet.

In conclusion, luteolin and apigenin, which do not belong to documented direct 4-HNE scavengers, protect PC12 cells from 4-HNE-induced cell death. Their cytoprotective effects were likely associated with modulation of 4-HNE-mediated UPR and resulted in increased expression of anti-apoptotic GRP78 and down-regulation of pro-apoptotic CHOP, TRB3 and GADD34. Luteolin also mitigated 4-HNE-mediated LC3 conversion and reactive oxygen species (ROS) production. Furthermore, pre-treatment of cells with luteolin or apigenin induced modest expression of xCT and HO-1, which in turn conferred inhibition to 4-HNE-induced sustained overexpression of HO-1 and xCT, indicating oxidative stress was repressed. Moreover, luteolin and apigenin enhanced p38 MAPK activation, which may confer an adaptive response to modulate 4-HNE-mediated stress responses and cytotoxicity (**Fig 8**).

## Supporting Information

S1 Fig Effect of rapamycin on 4-HNE-mediated cell death. PC12 cells were treated with indicated concentration of rapamycin alone or in combination with 4-HNE (25 μM) for 16 h at 37°C. Cell viability was measured by MTT as described in Materials and Methods. Data represent the mean ± SD of three independent experiments. **, *p*<0.01 represents significant differences compared with vehicle control (without 4-HNE). ##, *p*<0.01 represent significant differences compared with 4-HNE-treated vehicle group.
(DOCX)

S2 Fig Effects of luteolin and apigenin on rapamycin-induced cell death. PC12 cells were treated with vehicle (0.1% DMSO), luteolin or apigenin (10 and 20 μM) 30 min prior to rapamycin (0.1 μM) treatment for 16 h at 37°C. Cell viability was measured by MTT as described in Materials and Methods. Data represent the mean ± SD of three independent experiments. **, *p*<0.01 represents significant differences compared with vehicle control (without rapamycin). ##, *p*<0.01 represent significant differences compared with rapamycin-treated vehicle group.
(DOCX)

S3 Fig 4-HNE induced Nrf2 mRNA expression and Nrf2 protein nuclear translocation. (A) PC12 cells were treated with vehicle (0.1% DMSO), 4-HNE plus vehicle or 4-HNE plus 1 mM NAC. After 4 h, RNA was prepared and the mRNA expression of Nrf2 was analyzed by RT-Q-PCR and normalized to β-actin, as described in Materials and Methods. (B) After 6 h, nuclear cell lysates were prepared by Nuclear Extraction Kit (Cayman) and subjected to Western blotting analysis of Nrf2 and lamin A/C, which was used as a loading control. The blots are representative from one of three independent experiments. Data obtained from immunoblots were then analyzed using Phoretix Gel Analysis Software as described under Materials and methods. Data represent the mean ± SD of three independent experiments. **, *p*<0.01 represents significant differences compared with vehicle control (without 4-HNE). ##, *p*<0.01 represents significant differences compared with 4-HNE-treated vehicle group.
(DOCX)

S4 Fig Effects of luteolin and apigenin on tunicamycin-mediated cytotoxicity and unfolded protein responses. PC12 cells were treated with luteolin or apigenin (20 μM) 30 min prior to 4-HNE (25 μM) treatment at 37^°^C. (A) After 16 h, cell viability was measured by MTT as described in Materials and Methods. (B-F) after 4 h treatement, RNA was prepared and XBP1 splicing and the expression of CHOP, TRB3 and GADD34 was analyzed as described in Materials and Methods. The data represent the mean ± SD of three independent experiments. **p<0.01 represents significant differences compared with vehicle control (without 4-HNE); #*p*<0.01; ##, *p*<0.01 represent significant differences compared with the 4-HNE-treated vehicle group.
(DOCX)

S5 Fig Effect of H89, a PKA inhibitor, on 4-HNE-mediated cytotoxicity and expression of unfolded protein response and oxidative stress-related genes. (**A**) PKA inhibitor H89 attenuates the cytoprotective effects of luteolin and apigenin. PC12 cells were pretreated for 30 min with H89 (10 μM) and 20 μM luteolin or apigenin was then added 30 min prior to 4-HNE (25 μM) exposure for 16 h. MTT was used to analyze the cell viability. * *p*<0.05; **p<0.01 represent significant differences compared with respective no inhibitor group; #*p*<0.01; ##, *p*<0.01 represent significant differences compared with the 4-HNE-treated vehicle group. (**B-D**) H89 enhances 4-HNE-mediated CHOP, HO-1 and xCT expression. PC12 cells were pretreated for 30 min with H89 (10 μM) and 20 μM luteolin or apigenin was then added 30 min prior to 4-HNE (25 μM) exposure for 4 h. RNA was prepared and the expression of CHOP, HO-1 and xCT was analyzed by RT-Q-PCR and normalized to β-actin, as described in Materials and Methods. The data represent the mean ± SD of three independent experiments. * *p*<0.05; **p<0.01 represent significant differences compared with respective no inhibitor group; ##, *p*<0.01 represents significant differences compared with the 4-HNE-treated vehicle group.
(DOCX)
