# Supplementary material for: Luteolin and Apigenin Attenuate 4-Hydroxy-2-Nonenal-Mediated Cell Death through Modulation of UPR, Nrf2-ARE and MAPK Pathways in PC12 Cells
Source: PLoS One. 2015 Jun 18;10(6):e0130599. doi: 10.1371/journal.pone.0130599 (PMC4472230; doi:10.1371/journal.pone.0130599)
Supplement: S1 Fig — PC12 cells were treated with indicated concentration of rapamycin alone or in combination with 4-HNE (25 μM) for 16 h at 37°C. Cell viability was measured by MTT as described in Materials and Methods. Data represent the mean ± SD of three independent experiments. **, p<0.01 represents significant differences compared with vehicle control (without 4-HNE). ##, p<0.01 represent significant differences compared with 4-HNE-treated vehicle group. (DOCX) [file pone.0130599.s001.docx]

**S1 Fig.**
