# Supplementary material for: Luteolin and Apigenin Attenuate 4-Hydroxy-2-Nonenal-Mediated Cell Death through Modulation of UPR, Nrf2-ARE and MAPK Pathways in PC12 Cells
Source: PLoS One. 2015 Jun 18;10(6):e0130599. doi: 10.1371/journal.pone.0130599 (PMC4472230; doi:10.1371/journal.pone.0130599)
Supplement: S3 Fig — (A) PC12 cells were treated with vehicle (0.1% DMSO), 4-HNE plus vehicle or 4-HNE plus 1 mM NAC. After 4 h, RNA was prepared and the mRNA expression of Nrf2 was analyzed by RT-Q-PCR and normalized to β-actin, as described in Materials and Methods. (B) After 6 h, nuclear cell lysates were prepared by Nuclear Extraction Kit (Cayman) and subjected to Western blotting analysis of Nrf2 and lamin A/C, which was used as a loading control. The blots are representative from one of three independent experiments. Data obtained from immunoblots were then analyzed using Phoretix Gel Analysis Software as described under Materials and methods. Data represent the mean ± SD of three independent experiments. **, p<0.01 represents significant differences compared with vehicle control (without 4-HNE). ##, p<0.01 represents significant differences compared with 4-HNE-treated vehicle group. (DOCX) [file pone.0130599.s003.docx]

A


B

**S3 Fig.**
