# Supplementary material for: Luteolin and Apigenin Attenuate 4-Hydroxy-2-Nonenal-Mediated Cell Death through Modulation of UPR, Nrf2-ARE and MAPK Pathways in PC12 Cells
Source: PLoS One. 2015 Jun 18;10(6):e0130599. doi: 10.1371/journal.pone.0130599 (PMC4472230; doi:10.1371/journal.pone.0130599)
Supplement: S4 Fig — PC12 cells were treated with luteolin or apigenin (20 μM) 30 min prior to 4-HNE (25 μM) treatment at 37°C. (A) After 16 h, cell viability was measured by MTT as described in Materials and Methods. (B-F) after 4 h treatement, RNA was prepared and XBP1 splicing and the expression of CHOP, TRB3 and GADD34 was analyzed as described in Materials and Methods. The data represent the mean ± SD of three independent experiments. **p<0.01 represents significant differences compared with vehicle control (without 4-HNE); #p<0.01; ##, p<0.01 represent significant differences compared with the 4-HNE-treated vehicle group. (DOCX) [file pone.0130599.s004.docx]

**S4 Fig.**
