# Supplementary material for: Luteolin and Apigenin Attenuate 4-Hydroxy-2-Nonenal-Mediated Cell Death through Modulation of UPR, Nrf2-ARE and MAPK Pathways in PC12 Cells
Source: PLoS One. 2015 Jun 18;10(6):e0130599. doi: 10.1371/journal.pone.0130599 (PMC4472230; doi:10.1371/journal.pone.0130599)
Supplement: S5 Fig — (A) PKA inhibitor H89 attenuates the cytoprotective effects of luteolin and apigenin. PC12 cells were pretreated for 30 min with H89 (10 μM) and 20 μM luteolin or apigenin was then added 30 min prior to 4-HNE (25 μM) exposure for 16 h. MTT was used to analyze the cell viability. * p<0.05; **p<0.01 represent significant differences compared with respective no inhibitor group; #p<0.01; ##, p<0.01 represent significant differences compared with the 4-HNE-treated vehicle group. (B-D) H89 enhances 4-HNE-mediated CHOP, HO-1 and xCT expression. PC12 cells were pretreated for 30 min with H89 (10 μM) and 20 μM luteolin or apigenin was then added 30 min prior to 4-HNE (25 μM) exposure for 4 h. RNA was prepared and the expression of CHOP, HO-1 and xCT was analyzed by RT-Q-PCR and normalized to β-actin, as described in Materials and Methods. The data represent the mean ± SD of three independent experiments. * p<0.05; **p<0.01 represent significant differences compared with respective no inhibitor group; ##, p<0.01 represents significant differences compared with the 4-HNE-treated vehicle group. (DOCX) [file pone.0130599.s005.docx]

**S5 Fig.**
